# Deriving Movement Categories in Rugby Sevens

**DOI:** 10.1002/ejsc.70101

**Published:** 2025-12-23

**Authors:** Ciara Finnegan, Michael Scriney, Anna Donnla O’Hagan, Laura McManus, Orlaith Curran, Jane C. Walsh, Marija Bezbradica

**Affiliations:** ^1^ School of Health and Human Performance Dublin City University Dublin Ireland; ^2^ School of Computing Dublin City University Dublin Ireland; ^3^ Insight Centre for Data Analysis Dublin City University Dublin Ireland; ^4^ Irish Rugby Football Union High Performance Centre Dublin Ireland; ^5^ School of Psychology University of Galway Galway Ireland; ^6^ Adapt Research Centre Dublin City University Dublin Ireland

**Keywords:** global positioning systems, machine learning, rugby football, team sport, velocity thresholds

## Abstract

The primary aim of this study was to generate sport‐specific movement category velocity thresholds for elite rugby sevens male and female players. Match activity data were collected via Global Positioning Systems (GPS) (10 Hz) from 19 male and 11 female players during 88 competitive international fixtures during the 2022/2023 and 2023/2024 seasons. A two‐stage unsupervised clustering method was applied. The elbow method, a technique used to determine the optimal number of clusters in a dataset, was first applied to identify the number of movement categories. Spectral clustering was then used to define the velocity thresholds corresponding to each category. For both male and female rugby sevens, four movement categories were identified with varying velocity thresholds. The male movement category velocity thresholds were low (0.0–2.05 m.s^−1^), moderate (2.06–4.26 m.s^−1^), high (4.27–7.20 m.s^−1^) and very high (> 7.20 m.s^−1^). Although the female movement category velocity thresholds were low (0.0–1.87 m.s^−1^), moderate (1.88–3.74 m.s^−1^), high (3.75–5.97 m.s^−1^) and very high (> 5.97 m.s^−1^). A comparison of the total distance covered in the respective gender‐specific zones revealed that females covered a significantly less distance in the low‐velocity movement category (*p* = 0.02) and a significantly more distance in the very‐high‐velocity movement category (*p* < 0.001). This work informs customised movement categories that allow for better physical load assessments in male and female rugby sevens and the provision of sport‐specific and gender‐specific conditioning programmes.

## Highlights

- This research implemented spectral clustering to identify four sex‐ and sport‐specific movement categories for male and female rugby sevens during elite competition tournaments.
- Velocity thresholds differed between male and female players across all four movement categories, with males consistently reporting higher velocities. These increased thresholds align with the significant difference reported in maximum speed between male and female players.
- Male movement categories were categorised as low (0.0–2.05 m.s^−1^), moderate (2.06–4.26 m.s^−1^), high (4.27–7.20 m.s^−1^) and very high (> 7.20 m.s^−1^). Although the female movement categories’ velocity thresholds were low (0.0–1.87 m.s^−1^), moderate (1.88–3.74 m.s^−1^), high (3.75–5.97 m.s^−1^) and very high (> 5.97 m.s^−1^).

## 1 Introduction

Activity monitoring and the use of tracking systems have become routine practice in elite field‐based team sports (Torres‐Ronda et al. 2022). Common metrics often reported in relation to activity monitoring include total distance, relative distance, distance in velocity zones, maximum velocity, accelerations, decelerations, impacts and collisions (Torres‐Ronda et al. 2022; Ball et al. 2019).

Rugby sevens is a fast‐paced team sport that is characterised by periods of high‐intensity intermittent activity and collisions (Ross et al. 2014). After the establishment of the HSBC World Sevens Series in 1999, men's rugby sevens became a professionally recognised sport, whereas women's rugby sevens followed suit in 2012 (Rugby Sevens 2023). The game itself is a variant of rugby football, mirroring the rules and regulations of rugby union with small differences regarding set plays due to the reduced playing numbers (7 on‐field players per team) and match duration (7 min per half) (Ross et al. 2014).

Match demands and activity profiles of rugby sevens players are reported regularly within research (Sella et al. 2019). Differences between male and female players exist regarding these match demands (Ball et al. 2019). According to Clarke and colleagues (Clarke et al. 2017), international‐level male players cover a total distance of 1249 ± 348m and a relative distance of 103 ± 9 m.min^−1^. In comparison, female international players cover 1078 ± 197m total distance and 85.5 ± 3.9 m.min^−1^ relative distance. Differences in maximum velocity between male and female players also exist (male: 8.7 m.s^−1^, female: 8.1 m.s^−1^) (Clarke et al. 2017). Similarly, differences in distance covered across various velocity zones were reported. Male players covered greater distances at high‐speed velocities (HSR) (≥ 5 m.s^−1^) compared to female players (male: 190.3 m, female: 129.9 m). According to Ball and colleagues (Ball et al. 2019), at velocities > 3.5 m.s^−1^ male players cover 449.3 m, whereas female players cover 339.5 m (Ball et al. 2019). These velocity thresholds are reflective of early classifications developed in 1991 using manual video analysis (Park et al. 2019). Originating from Bangsbo et al. (1991), arbitrary velocity zones were developed for movement categories including standing, walking, jogging, running and sprinting based on mean squad velocities. However, these video‐derived thresholds lack physiological validation (Bangsbo et al. 1991). Despite advances in microtechnology and the accuracy, reliability and validity of these sensors (Hoppe et al. 2018), these classifications remain widely used in current practice (Higham et al. 2012, 2016; Suarez‐Arrones et al. 2012; Vescovi and Goodale 2015).

Differences in physiological capacity between males and females can inaccurately describe the movement demands of female players when quantified using thresholds specific to males (Bradley and Vescovi 2014). Prior research suggests that female HSR is frequently underestimated when utilising male‐based or arbitrary velocity thresholds derived from VO_2_ max, underscoring the necessity for gender‐specific thresholds (Clarke et al. 2015a). Similarly, in rugby union, when comparing positions, default velocity thresholds significantly underestimate HSR for forwards and overestimate HSR for backs when adjusted using the teams’ average max velocity (Reardon et al. 2015). However the requirement for max testing in season is viewed as an impracticality by practitioners due to scheduling, rendering it difficult to develop these physiologically defined movement categories (Bennett et al. 2023).

An alternative approach to the development of velocity thresholds is the application of advanced data analytics (Clemente et al. 2023). Recent advancements in this area, particularly the application of unsupervised machine learning, have created new opportunities to analyse movement categories in team sports. Unsupervised learning refers to a machine learning methodology that operates on unlabelled data, allowing the model to independently identify patterns and structures within the data (Horvat and Job 2020). Unlike traditional models that rely on fixed thresholds or group averages, these applied methods allow for the identification of movement categories based on the actual distribution of velocity data captured during competition via Global Positioning Systems (GPS) (Park et al. 2019; Brady et al. 2021). Based on a measure of similarity, unsupervised algorithms organise data into distinct groups known as ‘clusters’ (Ofoghi et al. 2013). One such method, ‘spectral clustering’, is a graph‐based approach used to cluster multivariate data into distinct groups by identifying strongly connected data points within the same cluster while minimising connections between points in a different cluster (Von Luxburg 2007). Park et al. (2019) were the first to apply the unsupervised clustering technique to match activity data, focusing on the international female soccer. Previous work in the area of data mining techniques saw the application of *k*‐means algorithms in identifying movement sequences in netball (Sweeting et al. 2017). Although this was successful in identifying specific movements such as walking with straight movement, the application to movement categories is less appropriate compared to spectral clustering (Park et al. 2019). Applications of this approach exist in elite female soccer (Park et al. 2019) and Gaelic football and hurling referees (Brady et al. 2021). In soccer, 4 unique velocity categories were generated, whereas 6 unique movement categories were generated for referees (Brady et al. 2021). With both the soccer study and the referee study providing logical validity for the results (Park et al. 2019; Brady et al. 2021), these findings highlight the context‐sensitive nature of data‐driven threshold development, with the number and structure of movement categories varying according to the population and sport being analysed. In rugby league, this approach also generated 4 velocity categories; however, the resulting thresholds differed from those identified in soccer despite both analyses being conducted on female players (Cummins et al. 2023). This may be attributed to variations in player capabilities and the sport‐specific requirements of rugby league relative to football (Datson et al. 2017; Emmonds et al. 2020). Therefore, the use of unsupervised clustering supports the development of sport‐specific or cohort‐specific movement categories that more accurately reflect the competition demands.

Identifying these zones will lead to more precise physical load monitoring, facilitating both gender‐specific and sport‐specific conditioning programmes. These customised movement categories will better replicate the intensity and demands of rugby sevens, potentially improving training effectiveness and performance outcomes. Therefore the primary aim of this study is to generate sport‐specific movement category velocity thresholds for elite rugby sevens male and female players using a two‐stage unsupervised clustering technique applied to global positioning data. The objective of this work is to compare the difference between male‐ and female‐generated movement categories. A second objective is to quantify and compare the distance covered in the respective movement categories between genders. It is hypothesised that differences between male and female rugby sevens players will exist regarding the movement category velocity thresholds generated.

## 2 Methods

### 2.1 Participants and Data

Data were obtained from 19 male and 11 female elite international rugby sevens players. All participants were active members of internationally competing teams. The dataset included 55 match files from male players and 33 match files from female players, collected retrospectively via GPS during competitive fixtures from the 2022/2023 and 2023/2024 seasons. Only players who completed the full duration of a match were included in the analysis. Positional data were not considered. Ethical approval was obtained from the Institutional Research Ethics Committee (DCUREC/2023/021).

### 2.2 Methodology

During each match, players wore 10‐Hz GPS devices (STATSports, Newry, Ireland). In comparison to GPS devices sampling at lower frequencies, the 10‐Hz device produces the most reliable data (Akenhead et al. 2014). The GPS device was worn between the shoulder blades in a specific custom‐made vest. The devices were activated a minimum of 30 min before the start of each match to ensure a GPS satellite lock (Malone et al. 2017). Data from the GPS device were downloaded into STATSports software before exporting the raw files into the Python (v.3.7) programming language (Python Software Foundation, Wilmington, DE, USA) for data cleaning and analysis.

Data cleaning involved the exclusion of data points if instantaneous velocity was > 10 m.s^−1^ (Park et al. 2019). Match files with a resulting > 3% of data points removed were excluded from the analysis. The resulting dataset consisted of 88 match files with an average of 2.9 matches per player. This resulted in a dataset consisting of 88 full matches (55 male, 33 female). There is an average of 2.9 matches per male player (range: 1–15 matches). For females, there is an average of 3.0 matches per player (range: 1–9 matches).

### 2.3 Unsupervised Clustering Technique

Movement category velocity thresholds were generated using an unsupervised learning technique, which was conducted independently for male and female rugby sevens players. Spectral clustering was employed to discretise the continuous velocity data into categorical variables by identifying transition points corresponding to the minimal number of traversals (Brady et al. 2021). This method has been previously outlined in research in soccer (Park et al. 2019) and Gaelic football and hurling referees (Brady et al. 2021), in which a traversal is defined as a change in velocity across adjacent timepoints. The velocity range for these traversals ranged between 0 m.s^−1^ and 10 m.s^−1^, with a width of 0.1 m.s^−1^, providing a total of 100 velocity ‘bins’. This range was computed and analysed using the spectral clustering algorithm. Spectral clustering operates by treating individual velocity values as distinct categories, disregarding their ordinal relationship, which may result in the grouping of 1.0 m.s^−1^ and 7.0 m.s^−1^ (Park et al. 2019). To address this limitation, a *β*‐coefficient of 0.1 was applied as a smoothing factor, preventing the clustering of velocity values that lack connected edges, as recommended in previous studies (Park et al. 2019; Brady et al. 2021).

Identification of the number of clusters *k* within the dataset was determined. The traversals of one individual file from a match were merged to form a new dataset. Spectral clustering was applied to this dataset for various values of *k* (Torres‐Ronda et al. 2022; Ball et al. 2019; Ross et al. 2014; Rugby Sevens 2023; Sella et al. 2019; Clarke et al. 2017; Park et al. 2019; Bangsbo et al. 1991; Hoppe et al. 2018; Higham et al. 2012). The elbow method was utilised in order to identify the number of categories (Wang et al. 2018). This method uses the within‐cluster sum of squares for each respective value of *k.* The resulting plot is used to identify the point of inflection, ‘the elbow’, which indicates the most appropriate value or number of clusters or categories. Once the optimal value of *k* was determined, identifying the corresponding velocity thresholds for each of the *k* clusters was completed. This method was applied to each match file using the predetermined value of *k*. Velocity thresholds were formed using the cohort mean value from the respective games.

### 2.4 Statistical Analysis

Statistical analysis was conducted using Python programming with relevant libraries including pandas, scipy and statsmodels implemented. Absolute (*m*) and relative (m·min^−1^) distances covered within each movement category were calculated using the 10‐Hz GPS data. Results are presented as mean ± standard deviation (SD). Differences between male and female players were assessed using independent‐samples *t*‐tests when data were normally distributed and Mann–Whitney U tests when the assumption of normality was not met. Additionally, the distances covered in each movement category using the generated velocity thresholds were compared to those derived from generic thresholds using independent‐samples *t*‐tests. These new movement categories were then compared to default arbitrary thresholds used in international rugby sevens (standing/walking; 0.0–2.0 m.s^−1^, jogging; 2.0–3.5 m.s^−1^, cruising; 3.5–5.5 m.s^−1^, high‐speed running; 5.5–6.0 m.s^−1^, sprinting; > 6.0 m.s^−1^) (Higham et al. 2016; Clarke et al. 2015b).

## 3 Results

### 3.1 Movement Categories

The number of movement categories, determined by the value of *k* corresponding to the inflection point, was equal to 4 for both male and female rugby sevens players. Table 1 displays the mean velocity for each of these movement categories.

**TABLE 1:** Movement categories of male and female rugby sevens players.

	Male	Female
Zone 1 (m.s^−1^)	< 2.05	< 1.87
Zone 2 (m.s^−1^)	≥ 2.05–4.26	≥ 1.87–3.74
Zone 3 (m.s^−1^)	≥ 4.27–7.20	≥ 3.75–5.97
Zone 4 (m.s^−1^)	> 7.20	> 5.97

### 3.2 Activity Profile

Male rugby sevens players covered an average total distance of 1378.63 ± 407.35 m per match, whereas female players covered 1345.78 ± 353.97 m per match (see Table 2). No significant difference was observed between male and female players in total distance covered. The relative distance covered per minute was 70.32 ± 19.92 m.min^−1^ for male players and 71.35 ± 14.81 m.min^−1^ for female players. Consistent with the findings for total distance, no significant difference was detected between males and females in relative distance covered per match. Male players achieved an average maximum velocity of 8.34 ± 1.03 m.s^−1^ compared to 7.40 ± 0.89 m.s^−1^ for female players. This difference in maximum velocity was statistically significant (*p* < 0.001).

**TABLE 2:** Activity profile of male and female rugby sevens players.

	Male	Female	*p* value
Total distance (m)	1378.63 ± 407.35	1345.78 ± 353.97	0.692
Relative distance (m.min^−1^)	70.32 ± 19.92	71.35 ± 14.81	0.782
Max speed (m.s^−1^)	8.34 ± 1.03	7.40 ± 0.89	< 0.001^a^

*Note:* Data are presented as mean ± standard deviation.

^a^ Denotes significant difference of *p* < 0.05.

### 3.3 Differences Between Male and Female Across Newly Generated Movement Categories

**FIGURE 1:**
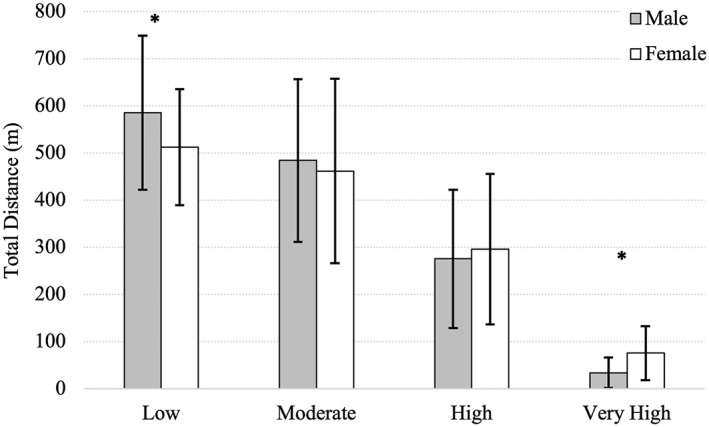
Distance covered within movement categories generated using spectral clustering for male and female rugby sevens players. Data are presented as mean with error bars representing standard deviation. * denotes significant difference of *p* < 0.05.

The total distances covered within the newly generated movement categories are presented in Table 3 and Figure 1. Significant sex‐based differences were observed in both the low‐velocity and very‐high‐velocity movement categories. In the low‐velocity category, female players covered significantly less distance than male players (*p* = 0.02; males: 585.40 ± 163.83 m, females: 512.57 ± 122.84 m). Conversely, in the very‐high‐velocity category, female players covered significantly greater distances compared to males (*p* < 0.001; females: 75.42 ± 57.13 m, males: 33.57 ± 32.13 m).

**TABLE 3:** Distance covered in each movement category for male and female rugby sevens players.

Zone	Male	Female	*p* value
Low	585.40 ± 163.83	512.57 ± 122.84	0.02^a^
Moderate	484.19 ± 172.74	461.76 ± 195.57	0.59
High	275.47 ± 146.84	296.02 ± 159.23	0.55
Very high (*μ*)	33.57 ± 32.13	75.42 ± 57.13	< 0.001^a^

*Note:* Data are presented as mean ± standard deviation. *μ* indicates use of Mann–Whitney *U* test.

^a^ Denotes significant difference of *p* < 0.05.

### 3.4 Differences Between Male and Female Across Generic Movement Categories

**FIGURE 2:**
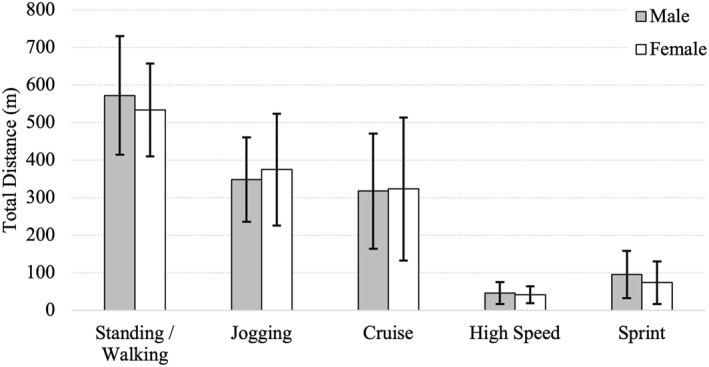
Distance covered within generic movement for male and female rugby sevens players. Data are presented as mean with error bars representing standard deviation.

A between‐gender comparison of total distance within the generic speed zones reported no significant difference across all 5 movement categories (see Table 4 and Figure 2). Males covered more total distance for standing/walking, high‐speed running and sprinting movement categories, whereas females performed more distance in the jogging and cruising movement categories.

**TABLE 4:** Distance covered in the generic movement category for male and female rugby sevens players.

Zone	Male	Female	*p* value
Standing/walking	572.25 ± 157.49	533.37 ± 123.88	0.20
Jogging	348.06 ± 111.91	374.66 ± 149.30	0.38
Cruise	317.38 ± 153.32	322.86 ± 190.33	0.89
High speed (*μ*)	45.86 ± 29.54	41.28 ± 22.00	0.65
Sprint (*μ*)	95.09 ± 63.19	73.61 ± 56.45	0.12

*Note:* Data are presented as mean ± standard deviation. *μ* indicates use of Mann–Whitney *U* test.

### 3.5 Differences Between Generated Movement Categories and Generic Movement Categories

**FIGURE 3:**
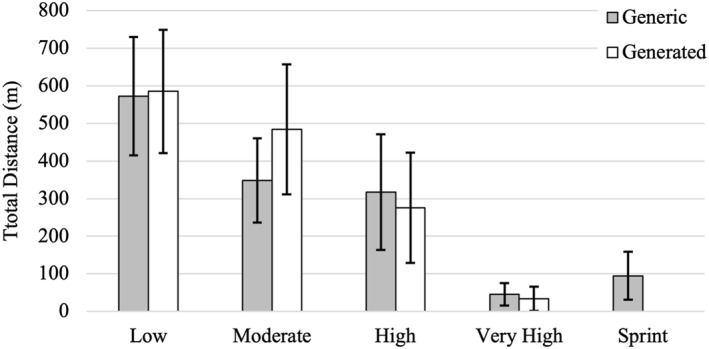
Male total distance in the generated movement categories and the generic movement categories. Data are presented as mean with error bars representing standard deviation.

**FIGURE 4:**
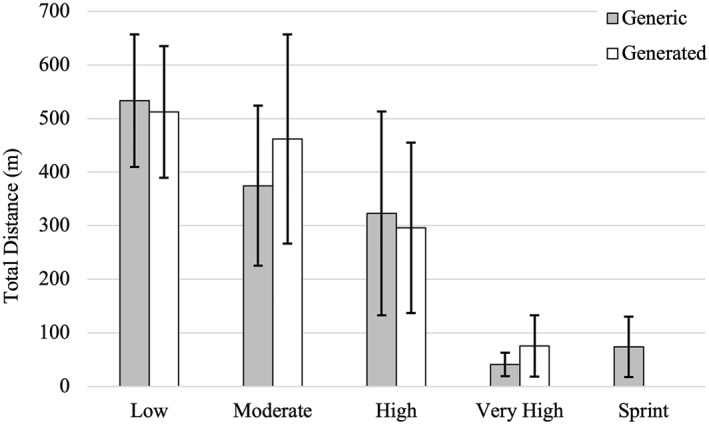
Female total distance in the generated movement categories and the generic movement categories. Data are presented as mean with error bars representing standard deviation.

Figures 3 and 4 illustrate the total distance covered by males and females, respectively, across both the generic and generated movement categories. After the determination of *k* and the generation of 4 movement categories, a direct comparison between generic and generated movement categories within genders was not possible. In order to create some comparison, a modification to the generic speed zones was performed. This modification combined the high‐speed running (5.5–6.0 m.s^−1^) and sprinting (> 6.0 m.s^−1^) zones in order to create 4 zones with total distances in the respective zones. For male players, the total distance in the low‐velocity movement category (585.4 ± 163.8 m) closely matched that of the standing/walking category (572.2 ± 157.5 m). Total distance in the moderate category (484.2 ± 172.7 m) was higher than in the generic jogging category (348.1 ± 111.9 m). The high‐velocity category showed slightly lower distances (275.5 ± 146.8 m) compared to the cruising category (317.4 ± 153.3 m). Although there was a large difference between the total distance covered in the very‐high‐velocity category (33.6 ± 32.1 m) and the combined high‐speed running and sprinting category (141.0 ± 85.0 m). Among female players, distances in the low‐velocity category (512.6 ± 122.8 m) were similar to that of the standing/walking velocity category (533.4 ± 123.9 m). The moderate‐velocity category (461.8 ± 195.6 m) yielded a higher total distance than the jogging velocity category (374.7 ± 149.3 m). The high‐velocity category total distance was slightly lower (296.0 ± 159.2 m) than the cruising velocity category (322.9 ± 190.3 m). As with males, the very‐high‐velocity movement category was less than the combined high‐speed running and sprinting velocity category.

## 4 Discussion

The primary aim of this study was to generate movement category thresholds for male and female elite international rugby sevens players. This was performed using an unsupervised clustering technique known as spectral clustering, which identified 4 movement categories for both male and female players. The movement categories were compared between male and female players regarding total distance across the categories while also being compared to movement category velocity thresholds previously reported in the literature. The results revealed significant differences between males and females regarding max velocity and the distance covered in the newly generated low‐velocity movement category and very‐high‐velocity movement category. It was not possible to compare the newly generated categories with the generic categories due to the difference in the number of movement categories. This is the first study of its kind to apply unsupervised clustering techniques to rugby sevens match play movement data in order to generate movement categories.

The application of spectral clustering is a growing trend in the analysis and discretisation of match activity data with applications in soccer (Park et al. 2019), Gaelic football and hurling (Brady et al. 2021) and rugby league (Cummins et al. 2023). The number of clusters identified in the international female soccer dataset corresponded to the results of this study, with 4 movement categories identified (Park et al. 2019). The movement category thresholds reported were low (0.0–3.46 m.s^−1^), moderate (3.47–5.29 m.s^−1^), high (5.30–6.26 m.s^−1^) and very high (> 6.26 m.s^−1^). A similar study completed in rugby league (Cummins et al. 2023) reported 4 distinct movement categories, again consistent with the findings of both the soccer study and this study. Despite the similar number of categories, the specific velocity thresholds themselves were different from those reported in this work. In rugby league, movement categories were classified as low (0.0–3.19 m.s^−1^), moderate (3.20–4.85 m.s^−1^), high (> 4.86 m.s^−1^) and very high (> 5.80 m.s^−1^), based on match play data from elite female players. In contrast, the velocity thresholds generated in this study were established for both male and female rugby sevens players using match play movement data from the respective cohorts. For female rugby sevens players specifically, the thresholds were lower in comparison to rugby league categories. These movement categories were classified as low (0.0–1.87 m.s^−1^), moderate (1.88–3.74 m.s^−1^), high (3.75–5.97 m.s^−1^) and very high (> 5.97 m.s^−1^). When comparing the total distances within the respective zones for rugby league (Cummins et al. 2023) and this study, sport‐specific differences are evident. In the study by Cummins et al. (2023), 79.1% of the total distance was completed at low speed compared to only 38.1% in rugby sevens. The distinct movement category velocity thresholds identified in each of the respective studies focusing specifically on females highlight the differences between sport and between rugby codes. Rugby league requires greater sustained movement intensities due to differences in positional roles, playing structures and match characteristics (Newans et al. 2021; Quinn et al. 2020). Similarly, the physical and tactical demands of soccer differ from those of rugby, influencing the generation of movement category velocity thresholds. Factors such as match duration, field dimensions and positional roles contribute to the distinct match demands of international soccer (Datson et al. 2017; Winther et al. 2022; Hewitt et al. 2014). Therefore, velocity thresholds derived for soccer are not directly transferable to rugby contexts, reinforcing the requirement for sport‐specific movement categories.

To date, the only known application of spectral clustering to activity data in male cohorts is the work of Brady et al. (2021), which focused on Gaelic football and hurling referees. This study identified 6 distinct movement categories in contrast to the 4 movement categories identified in this study. This discrepancy in the number of movement categories may be attributed to differences in cohort characteristics. Specifically, the referees’ population had an average age of 40.1 ± 4.5 years, which is in contrast to the younger age profile of a typical elite rugby sevens team (Sella et al. 2019). Referees also typically engage in more uniform movement patterns with less demand for explosive intensity (Brightmore et al. 2016), whereas rugby sevens players perform more high‐intensity movement (Higham et al. 2016; Ross et al. 2015). These contrasting physical demands and age‐related capacities likely impact the movement categories identified. This underscores the need for both population‐ and sport‐specific movement categories, warranting further research in this area of tailored movement category velocity thresholds in both male and female athletes across different sports, populations and competition levels.

The activity profile of both male and female rugby sevens players corresponds with results in previous studies (Ball et al. 2019; Clarke et al. 2017; Brosnan, Visentin et al. 2022). In this study, there was no significant difference between males and females regarding total distance or relative distance covered during match play. These results agree with an in‐depth meta‐analysis completed on the movement demands of rugby sevens players (Ball et al. 2019). At the international level, no differences were reported between male (1413.0 m) and female (1216.8 m). In this same meta‐analysis, no differences were reported between males and females regarding relative distance covered, again agreeing with the results of this study. According to the present findings, male players achieved a significantly greater maximum velocity than their female counterparts (male: 8.34 ± 1.03 m.s^−1^, female: 7.40 ± 0.89 m.s^−1^, *p < 0.001*). This finding coincides with the results in the meta‐analysis, with international male players achieving significantly greater max velocities than female players. This aligns with established physiological distinctions between genders, including differences in strength, power and speed (Hunter et al. 2023). These underlying gender differences likely contribute to the higher movement category velocity thresholds reported in this study. However, the differences may not only be attributed to differences in physiological capacity of males and females but also to differences in the rugby sevens matches themselves. A study examining match play characteristics among successful and unsuccessful rugby sevens teams identified gender‐based tactical differences (Barkell et al. 2016). Successful male teams were more likely to engage in a contact‐oriented style of play, characterised by a higher frequency of scrums and a greater tendency to kick to touch. In contrast, successful female teams demonstrated a preference for advancing towards the opposition try line through sustained passing sequences and more frequently utilised uncontested restarts (Barkell et al. 2016). These tactical distinctions suggest that male and female teams may approach the game differently, potentially influencing the types and intensities of movements performed during matches. Such findings underscore the importance of considering both physiological and tactical contexts when interpreting activity profiles in rugby sevens rather than relying solely on physiologically derived movement categories.

Physiologically defined movement categories are well documented within the literature (Clarke et al. 2015a; Reardon et al. 2015; Massard et al. 2022; Rojas‐Valverde et al. 2022; Rago et al. 2020). These movement categories are typically established using objective physiological measures to generate velocity thresholds. For example, movement categories have been developed based on speed at the second ventilatory threshold (VT_2speed_), a key indicator of aerobic capacity (Clarke et al. 2015a). Other approaches rely on the mean max velocity attained during match play or specific max velocity testing (Reardon et al. 2015; Massard et al. 2022; Rojas‐Valverde et al. 2022). Although, more recently, movement category velocity thresholds were developed through a combination of max aerobic speed, max sprinting speed and anaerobic speed reserve (Rago et al. 2020). These physiologically derived movement categories are valuable for assessing an athlete’s individual capacity and informing training intensities. However, their application is not without limitations. Conducting maximal aerobic or max velocity testing mid‐season is often challenging due to condensed competition schedules (Bennett et al. 2023). Furthermore, although these thresholds reflect individual capacity, they do not necessarily capture the contextual‐ or tactical‐specific demands of match play. Therefore, relying on physiologically defined movement categories may prove limited when analysing the in‐match demands of rugby sevens. This underscores the need for complementary approaches, such as data‐driven or match‐derived movement classifications, which can provide a more context‐specific understanding of external load and better reflect the demands placed on players during actual competition.

### 4.1 Limitations

The movement category velocity thresholds in this study reflect the specific demands of the sample cohort in male and female rugby sevens. Although not a focus of this work, the contextual factors surrounding the matches within the data, such as competition level, match status, opposition rank and tactical strategies employed, may influence the demands of the game. Hence, the movement categories identified are both sport‐specific and cohort‐specific. Although spectral clustering offers distinct advantages when applied to movement data, its application in team sports remains relatively infant. Future research should utilise larger sample sizes and focus across a range of sports and competition levels.

### 4.2 Practical Application

Using data mining techniques such as spectral clustering to generate movement categories unique to gender and sport offers substantial practical advantages in elite sport settings. Practitioners can gain an increased understanding of the physical demands imposed on athletes during competition by directly determining velocity thresholds from match data. Because the amount of time and distance spent in each velocity zone is more in line with the actual movement patterns seen in that specific sport and gender group, these customised classifications enable more accurate load monitoring. This enhances training transfer and performance results by supporting the development of focused conditioning regimens that replicate the specific needs and intensities experienced during match play.

## 5 Conclusion

This study effectively applied spectral clustering to identify sport‐specific movement category velocity thresholds for elite rugby sevens male and female players. To the best of the authors' knowledge, this is the first study that applies unsupervised clustering techniques to male and female rugby sevens match play data to generate and compare movement categories. Adding to the growing body of literature applying unsupervised clustering, this study identified 4 distinct movement categories for both males and females. Differences in the movement category velocity thresholds between male and female players highlight the physiological gender‐based differences and differences in match demands across male and female rugby sevens matches. It is recommended that activity data from rugby sevens matches be examined within these velocity thresholds for movement categories that are both sport specific and gender specific. Collectively, these results highlight the need for gender‐specific activity profiling in rugby sevens. These tailored movement category velocity thresholds can offer more accurate evaluations of the demands of the match in addition to more practical insights for conditioning, performance tracking and gender‐specific training recommendations.

## Funding

This work was conducted with the financial support of the Science Foundation Ireland Centre for Research Training in Digitally Enhanced Reality (d‐real) under Grant No. 18/CRT/6224.

## Ethics Statement

This study was conducted in accordance with the ethical standards of the Dublin City University and with the 1964 Helsinki Declaration and its later amendments. Ethical approval was obtained from the Dublin City University Research Ethics Committee (DCUREC/2023/021).

## Consent

This study involved the retrospective analysis of previously collected and fully anonymised data. As such, it did not involve any direct interaction with human participants, and informed consent was not required.

## Conflicts of Interest

The authors, Ciara Finnegan, Michael Scriney, Anna Donnla O'Hagan, Laura McManus, Jane C. Walsh, Orlaith Curran and Marija Bezbradica, declared no potential conflicts of interest with respect to the research, authorship and/or publication of this article.

## Data Availability

The data that support the findings of this study are available upon request from the corresponding author. The data are not publicly available due to privacy or ethical restrictions.
